# Therapeutic Effects of Probiotic *Pediococcus acidilactici* CNCM MA 18/5 (Bactocell PA) via Modulation of Oxidative Stress and Inflammatory Pathways in Streptozotosin Induced Diabetic Rats

**DOI:** 10.1002/fsn3.71902

**Published:** 2026-05-21

**Authors:** Betul Apaydin Yildirim, Enes Tercanli, Kubra Asena Terim Kapakin, Tuba Karaarslan, Mustafa Atasever

**Affiliations:** ^1^ Department of Biochemistry, Faculty of Veterinary Medicine Atatürk University Erzurum Türkiye; ^2^ Department of Food Hygiene and Technology, Faculty of Veterinary Medicine Atatürk University Erzurum Turkiye; ^3^ Department of Pathology, Faculty of Veterinary Medicine Atatürk University Erzurum Türkiye

**Keywords:** antioxidant, diabetes mellitus, oxidative stress, *Pediococcus acidilactici*, probiotic

## Abstract

Diabetes remains a global public health concern. This study aimed to investigate the therapeutic effects of *Pediococcus acidilactici* CNCM MA 18/5M (PA) against diabetes. Animals were randomly divided into 5 groups. Diabetes mellitus (DM) was induced in rats by administration of a single dose of 60 mg/kg of streptozotocin intraperitoneally. Animals were fed 1 mL of milk fermented with PA (1 × 10^8^ CFU/mL) for 20 days. Blood and pancreatic tissue were obtained from the anesthesia‐induced sacrifice of all rats. The levels of serum glucose, HbA1c, pancreatic tissue MDA, TNF‐α, and IL‐1β were increased in the diabetes group when compared with the control and DM group, while serum insülin level, CAT, and SOD activities were lower in the diabetes group. Administration of PA in rats with DM decreased glucose, HbA1c, pancreatic tissue MDA, TNF‐α, and IL‐1β levels, increased serum insülin level; CAT and SOD activities. PA reduced below the level of expression, the genes NF‐κB and TLR‐4, whereas HO‐1, Nrf‐2, and Keap‐1 were elevated. Based on biochemical parameters and histopathological findings, PA, as determined in preliminary studies, effectively lowered blood glucose and HbA1c levels in diabetic rats, reduced lipid peroxidation, enhanced antioxidant enzyme activity, and mitigated oxidative stress. Furthermore, since no prior research has investigated the impact of *Pediococcus acidilactici* CNCM MA 18/5 on NF‐κB, Tlr‐4, and the HO‐1, Nrf‐2, and Keap‐1 signaling pathways in STZ‐induced diabetic pancreatic tissue, this study serves as a pioneering contribution to the literature.

## Graphical abstract

In streptozotocin‐induced diabetic rats, the probiotic *Pediococcus acidilactici* CNCM MA 18/5 (Bactocell PA) reduced hyperglycemia, oxidative stress, and inflammatory responses. When PA was administered, insulin levels increased, glucose and HbA1c concentrations decreased, antioxidant enzyme activities (SOD and CAT) were elevated, and pro‐inflammatory cytokines (TNF‐α and IL‐1β) and lipid peroxidation were inhibited. Additionally, PA altered the HO‐1/Nrf‐2/Keap‐1 and NF‐κB/TLR‐4 signaling pathways, suggesting its potential as a therapeutic agent for diabetes.

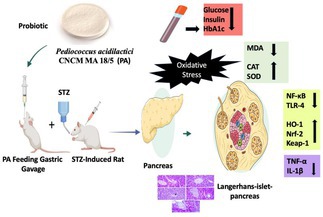

## 1 Introduction

Diabetes Mellitus (DM) is a growing public health concern, with its prevalence rising significantly worldwide over the past decade. In 2024, diabetes will impact over 589 million persons globally, and 1.1 billion adults suffer from impaired glucose tolerance or impaired fasting glycaemia. Over the next 20 years, there will likely be a large increase (IDF 2025). Despite the abundance of information currently available on diabetes treatment and prevention, the prevalence of type 2 diabetes is still rising worldwide. The vast majority of people who could benefit from optimal diabetes management are not receiving it. T2DM patients frequently have poor glycaemic control, which falls short of the WHO's target of 80% of those with diabetes having good glycaemic control (WHO 2025). It is widely recognized that insulin resistance—driven by negative lifestyle changes such as inadequate physical activity and poor dietary habits—as well as genetic predisposition, play key roles in its pathogenesis. However, recent evidence highlights the influence of environmental factors, particularly the gut microbiota, in diabetes development. Studies have shown that alterations in the gut microbiota disrupt bacterial balance, leading to increased intestinal permeability, reduced blood levels of Glucagon Like Peptide 1 (GLP‐1) and GLP‐2, and elevated endotoxin levels. These hormonal changes and endotoxemia contribute to diabetes by promoting lipogenesis, fat accumulation, inflammation, and macrophage infiltration in target organs, while simultaneously reducing insulin sensitivity, β‐cell mass, and plasma insulin levels. Given this, a key therapeutic goal is to restore gut microbiota balance to prevent diabetes or mitigate its complications. Nutrition plays a critical role in microbiota regulation, and recent studies suggest that prebiotic and probiotic interventions can help restore intestinal flora. Prebiotics and probiotics have been shown to reduce inflammation in diabetic patients, regulate blood lipid profiles, support weight management, enhance antioxidant defenses, improve pancreatic β‐cell function and insulin sensitivity, and aid in blood glucose regulation (Kamarlı 2019).

Many oral anti‐diabetic drugs are commonly used to manage hyperglycemia; however, they are often associated with side effects. These include hypoglycemia, sore throat and leukopenia. In particular, insulin has been linked to cause weight gain and deadly hypoglycemia (Kalra et al. 2013). Metformin, a biguanide commonly used in clinical practice, enhances insulin action in the liver while reducing glucose output and improving glucose utilization in muscle tissue (Caballero 2003). However, hypoglycemic agents are known to have adverse effects on the gastrointestinal tract, including ketonuria, lactic acidosis and indigestion. The discovery of novel, affordable medicines with minimal side effects is essential given the serious negative effects of current anti‐diabetic medications. Recent researches have shown a connection between gut microbial dysbiosis in diabetic individuals and metabolic disruptions such as dyslipidemia and insulin resistance (Markowiak and Ślizewska 2017; Wang et al. 2021).

Probiotics exhibit hypoglycemic, antioxidant, and anti‐inflammatory properties, making them a promising antidiabetic agent (Khan et al. 2021). They are live microorganisms that, when given in the right amounts, can offer a variety of health advantages (Markowiak and Ślizewska 2017). Probiotic lactic acid bacteria are among the most prevalent probiotics found in fermented milk and dairy products. They don't create toxins or dangerous metabolites, hence they are typically regarded as nonpathogenic (Ayivi et al. 2020). Additionally, probiotics support gut barrier function by providing energy for epithelial growth and inhibiting microbial invasion through the production of short‐chain fatty acids (SCFAs) (Markowiak‐Kopeć and Śliżewska 2020).

Mounting evidence suggests that the gastrointestinal microbiota is a crucial environmental factor in the development of prediabetes and Type 2 Diabetes (T2DM). Changes in intestinal permeability, inflammation, metabolism, energy homeostasis and most importantly insulin resistance are among the suggested molecular connections (Gurung et al. 2020; Wang et al. 2021).

Research indicates that individuals with diabetes exhibit distinct microbiota characteristics. In particular, it has been discovered that individuals with diabetes have an enrichment of opportunistic microorganisms, a decrease in butyrate‐producing bacteria, and a reduced gene count (abundance). Several lines of evidence further suggest that gastrointestinal microbiota may influence both the onset and progression of T2DM. However, the causal relationship between gut dysbiosis and T2DM remains unclear (Delzenne et al. 2015).

A recent study found that individuals who later developed T2DM already exhibited a distinct gastrointestinal microbiota composition before experiencing glucose homeostasis dysregulation. Additionally, another study reported that gastrointestinal microbiota composition evolves over the course of T2DM, as individuals with PreD display microbiota features distinct from both healthy individuals and those with T2DM (Zhang et al. 2021). Moreover, GM plays a role in mediating the effects of antidiabetic drugs (Vallianou et al. 2019; Gurung et al. 2020). Human studies have shown that individuals undergoing pharmacological treatment exhibit different GM compositions compared to untreated individuals (Chávez‐Carbajal et al. 2020).

The *Pediococcus acidilactici* CNCM MA 18/5M (Bactocell PA) strain is a lactic acid bacterium approved by the European Food Safety Authority and is commercially available as a feed additive. *Pediococcus acidilactici* (PA) strains with probiotic properties are also recognized as food supplements by the European Food Safety Authority (EFSA) (Yıldırım and Tuncer 2022). These strains are commonly isolated from various fermented products, such as cheese, pickles, and wine. Additionally, *P. acidilactici* is widely used in the fermentation of sausages and vegetables in the food industry (Papagianni and Anastasiadou 2009). Dietary factors play a crucial role in modulating the gut microbiota and are also key contributors to the development of prediabetes and T2DM (Zheng et al. 2018). Consequently, various dietary strategies, including fermented foods, functional foods, postbiotics, and probiotics, are being explored for their potential to counteract diabetes‐associated dysbiosis and prevent or even reverse diabetic complications (Cabello‐Olmo et al. 2019, 2021).

Probiotics can modify gastrointestinal microbiota composition and activity, enhance gut mucosal integrity, regulate the immune system, and provide protection against pathogens (Rondanelli et al. 2017). Due to these beneficial effects, probiotics have been extensively studied in the context of various diseases and conditions (Sanders et al. 2018). Probiotics have been shown in trials on humans and animals with type 2 diabetes to enhance glycemic management, lower inflammation and insulin resistance, increase mucus production, and improve intestinal barrier function (Jafarabadi et al. 2020; Salles et al. 2020).

Since there are limited studies on the effects of Bactocell PA commonly used in aquaculture, pig, and poultry farming on diabetic rats, this study aimed to investigate the impact of diabetes on the pancreas through biochemical, molecular, and histopathological analyses and to evaluate the affected pathways.

## 2 Materials and Methods

### 2.1 Chemicals Used in the Examination

The Merck companies supplied the chemical components used in the investigation. According to the literature, Streptozotocin (STZ) (Sigma, St. Louis, MO, USA) was dissolved in a 0.1 M pH: 4.5 citrate buffer (cold) and administered intraperitoneally (60 mg/kg, i.p.) (Gilani et al. 2021; Yildirim et al. 2024). Metmorfin HCl (Matofin XR 500 mg, Sanovel, Türkiye) (Tercanlı 2024).

### 2.2 Bacterial Starter Cultures and Milk Fermentation

Probiotic milk was prepared by diluting commercially purchased skim milk powder with water at a 1:9 ratio, mixing it with skim cow's milk, and sterilizing it at 115°C for 15 min (Tercanlı 2024). *Pediococcus acidilactici* CNCM MA 18/5 M culture (Bactocell, Lallemand Animal Nutrition, France) was commercially obtained. The bacteria, in powder form, were purified by adding De Man‐Rogosa‐Sharpe agar (MRSA, Merck, Jakarta, Indonesia) supplemented with 0.5 g/L L‐cysteine (Sigma‐Aldrich, Panjang Rd., Singapore) under aseptic conditions and incubating at 37°C for 24 h. *P. acidilactici* strain were grown on MRS broth and harvested at logarithmic phase. Microorganisms were adjusted to 0.5 McFarland (10^8^ CFU) using Densichek Plus McF (bioMérieux, France) and inoculated into 250 mL of skim milk (Tercanlı 2024). The mixture was then incubated at 37°C and left to ferment for 20 h. Fresh probiotic milk was prepared daily for 21 days, and 1 mL was administered orally to the rats via gastric gavage (Tercanlı 2024).

### 2.3 Experimental Animals

The rats used in the study were provided by the Medical Experimental Application and Research Center (ATADEM), affiliated with Ataturk University, and were approved according to the required ethics committee document, dated 18.12.2024, with the letter number E‐36643897‐000‐2400417803. Thirty‐five male Sprague Dawley rats (6–8 weeks) weighing 200–250 g were split into five groups, each containing seven rats: the Control group (C), which did not have diabetes; the Diabetes+Metformin HCl group (DM+MET); the Diabetes group (DM); the Diabetes+ *Pediococcus acidilactici* group (DM+PA); and the *Pediococcus acidilactici* group (PA). Rats were divided into 5 groups by weighing and equalizing the average weights, and 7 animals were in each cage. Rats were fed ad libitum standard rat chow and tap water and were housed under controlled conditions (22°C ± 2°C temperature, 55% ± 5% humidity, 12:12 h light/dark cycle).

### 2.4 Experimental Design

After 18 h of fasting to induce diabetes (free access to water), STZ was administered intraperitoneally (ip) to the DM+MET, DM, and DM+PA groups, along with an equivalent volume of cold citrate buffer (0.1 M, pH = 4.5, 0.5 mL) ip. Rats were classified as diabetics if their fasting blood glucose level, as determined by a glucometer 7 days after STZ treatment, was 250 mg/dL or higher. PA was administered orally once daily for 20 days via gastric gavage to the DM+PA and PA groups (Yildirim et al. 2024; Onay et al. 2026). According to the literature, the DM+MET group received MET orally once daily for 20 days via gastric gavage at a dose of 45 mg/kg (Widodo et al. 2023). On the 28th day of the experiment, all rats were decapitated in order to collect samples of pancreatic tissue and blood using Xylazine (8 mg kg^−1^) and ketalar (60 mg kg^−1^) anesthetics. The obtained blood was put in vacuum tubes, centrifuged for 10 min at +4°C and 3000 rpm, and the serum was separated and stored at −20°C in a deep freezer before biochemical analysis. A section of the pancreas tissue was excised and immersed in 10% formaldehyde for histological analysis; the remaining piece was stored at −20°C in a deep freezer until biochemical examination (Yildirim et al. 2024, Onay et al. 2026).

### 2.5 Some Biochemical and Oxidative Stress Parameters

Serum insulin levels were then measured both before treatment initiation and at the end of the treatment using ELISA, following the manufacturer's instructions (Farino et al. 2016). Blood glucose levels were measured using Tinder's method (Lott and Turner 1975). Hemoglobin A1c (HbA1c) levels were recorded following the standard protocol described by John et al. (2014). Briefly, the HbA1c absorbance was measured at 415 nm after the reagent and blood were combined. Serum glucose, insulin, and HbA1c levels were measured using the Biotech Epoch UV–Visible ELISA spectrophotometer. For biochemical analysis, the required quantity of pancreatic tissue was weighed, appropriate buffer solutions were selected based on the analysis, diluted, and homogenized using Qiagen TissueLyser II. Pancreatic tissue TNF‐α (Cat. No: 201‐11‐0765, Shanghai SunRed Biological Technology Co. Ltd., China) and interleukin 1 beta (IL‐1β) (Cat. No: 201‐11‐0120, Shanghai SunRed Biological Technology Co. Ltd., China) were performed according to the protocol of the purchased kit. The Biotech Epoch UV–Visible ELISA spectrophotometer was used to measure the levels of pancreatic tissue TNF‐α, IL‐1β, MDA (Placer et al. 1966) level; CAT (Goth 1991) and SOD (Sun et al. 1988) activities. In order to express SOD activities in g protein, protein levels were assesed in pancreatic tissues (Lowry et al. 1951).

### 2.6 Western Blotting Analysis

The amount of protein in pancreatic tissue was determined using a method described in a previous study (Dogan et al. 2025; Onay et al. 2026). Approximately 40 mg of pancreatic tissue was weighed into pre‐chilled RIPA lysis buffer and homogenized to prepare the pancreatic tissue samples. The samples were centrifuged for 20 min at 16.000 G after homogenization. Following complete lysis and grinding, the samples were centrifuged to collect the supernatant, which was utilized to extract all of the proteins from the liver tissue. The BCA assay (Smith's Thermo PierceTM BCA measurement kit) was used to detect protein concentrations. In order to guarantee total protein denaturation in advance of gel electrophoresis, the samples were subsequently heated in a water bath at 100°C for 10 min. 10% SDS‐PAGE gel electrophoresis was used to load the produced samples. For protein transfer, the samples were transferred onto a PVDF nitrocellulose membrane that had been blocked with 5% skim milk powder. Following this, the membrane was washed with TBST (5 min). Primary antibodies against NF‐κB (1:1000; sc‐8008), (TLR‐4) (1:1000; sc‐293072), HO‐1 (1:1000; sc‐390991), Nrf‐2 (1:1000; sc‐365949), Keap 1 (1:1000; sc‐365626), and β‐tubulin (1:1000; sc‐47778) were used to probe the membranes that had been incubated overnight at +4°C for the whole night. Following this TBST wash and then the blots were incubated with Goat Anti‐Mouse IgG secondary antibodies (1:2000; sc‐2005, Santa Cruz Biotechnology, USA) at room temperature for 2 h. After the membrane was treated with the secondary antibodies, it was washed five times with TBST for 5 min each. The Enhanced Chemi Luminescence (Trident femto Western HRP Substrate, Cat.: GTX14698) luminescence was utilized to visualize protein bands, and the data were recorded using the Biorad Gel Doc XR Imaging System (BIO‐RAD, USA). ImageLab software from Bio‐Rad was utilized to perform a densitometric analysis of the bands in order to quantify the expression levels of the proteins. At least three more measurements were made for each sample.

### 2.7 Pancreatic Tissue Histopathological Analysis

Rats pancreatic tissues were fixed 10% buffered formalin for histopathology examinations. Pancreatic tissues taken for histological analysis were fixed in a 10% formalin solution for 24 h, followed by a 10‐h wash under running tap water. They underwent a succession of alcohol and xylol treatments before being embedded into paraffin blocks after routine tissue monitoring. Each block was sliced into slices that were 4 μm thick in order to create slides. Hematoxylin–eosin (HE) stains were used to staining of preparations made for histopathological examination, and examined using light microscope an Olympus Bx51 with DP72 camera system (Olympus Corp., Tokyo, Japan) (Terim Kapakin et al. 2024). Ten randomly selected from all pancreas tissue samples areas in each section were examined x20 dimension images were taken under a high‐power light microscope. Scores for inflammation based on degree of degenerative and necrotic changes in the islets of Langerhans were generated semiquantitatively. Briefly, 10 random fields/slide/rat were selected, examined blindly and scored as follows: (−) absence of the lesion (no staining) = 0%, (+) mild staining = 5%–25%, (++) moderate staining = 26%–50%, and (+++) severe damage (strong staining) ≥ 50% (Iskender et al. 2021; Onay et al. 2026).

### 2.8 Statistical Analysis

Data from all groups were analyzed using one‐way analysis of variance (ANOVA) conducted using IBM SPSS Statistics 2*8*.0 package program to compare statistical variations among different groups, Tukey's multiple comparison test. Measurement data was presented using mean ± standard error of the mean (SEM) to showcase the information. The value of *p* 0.05 was considered statistically significant for variances.

## 3 Results

### 3.1 Evaluation of Serum and Pancreatic Tissue Biochemical Parameters

Changes in some biochemical parameters of serum taken from rats as a result of experimental diabetes induced by streptozotocin, serum glucose, insulin, and HbA1c levels; pancreatic tissue TNF‐α, IL‐1β, MDA level, CAT and SOD activity are shown in Table 1.

**TABLE 1:** Some biochemical parameters measured in the plasma and pancreatic tissue of groups.

	*C*	DM+MET	DM	DM+PA	PA	p
Plasma						
Glucose (mg/dL)	84.86 ± 0.59^c^	89.43 ± 1.29^c^	377.86 ± 7.23^a^	124.86 ± 1.55^b^	82.86 ± 0.83^c^	***
Insulin (μLU/mL)	18.29 ± 0.18^a^	15.57 ± 0.20^c^	5.29 ± 0.29^e^	14.57 ± 0.20^d^	20.29 ± 0.18^b^	***
HbA1c (mg/g of Hb, %)	0.53 ± 0.02^c^	0.56 ± 0.02^c^	1.66 ± 0.02^a^	0.64 ± 0.02^b^	0.20 ± 0.01^d^	***
Pancreatic tissue						
TNF‐α (ng/L)	51.30 ± 0.75^c^	64.28 ± 2.70^b^	129.17 ± 1.66^a^	71.41 ± 2.22^b^	48.10 ± 1.66^c^	***
IL‐1β (pg/L)	171.15 ± 2.68^d^	210.60 ± 2.12^c^	530.54 ± 12.49^a^	247.94 ± 8.25^b^	151.93 ± 6.51^d^	***
MDA (nmol/g tissue)	14.96 ± 0.46^c^	19.21 ± 0.46^c^	130.28 ± 1.62^a^	29.67 ± 1.99^b^	14.63 ± 0.70^c^	***
CAT (kU/g tissue)	149.60 ± 2.19^b^	141.89 ± 0.90^b^	103.71 ± 2.19^d^	122.10 ± 1.89^c^	157.61 ± 4.12^a^	***
SOD (EU/g protein)	34.09 ± 2.39^ab^	35.85 ± 0.65ab	25.22 ± 0.95^c^	32.96 ± 0.75^b^	38.59 ± 0.31^a^	***

*Note:* ^a,b,c,d^Means within rows with different superscripts are significantly different.

Abbreviations: C, control group; DM, diabetes group; MET, metformin; PA, *Pediococcus acidilactici* group.

*** *p* < 0.001.

Regarding serum levels of glucose and HbA1c a substantial elevation was observed in the diabetic group compared to the control rats. However, rats treated with PA exhibited a significant reduction in these parameters relative to the diabetic group (*p* < 0.001). This elevation was alleviated considerably in the PA treatment groups when compared to the diabetic group. Additionally, while the diabetic group showed a marked decline in serum insulin levels, a significant increase was observed in the PA‐treated group (*p* < 0.001).

Compared to the control rats, diabetic rats exhibited a significant increase in pancreatic tissue levels of MDA, TNF‐α, and IL‐1β, along with a depletion of antioxidant biomarkers (CAT and SOD). However, treatment with PA significantly reversed these changes by increasing pancreatic tissue activities of CAT and SOD enzymes. Additionally, PA treatment reduced serum MDA levels while enhancing CAT and SOD activities (*p* < 0.001).

### 3.2 Evaluation of Western Blotting Analysis

**FIGURE 1:**
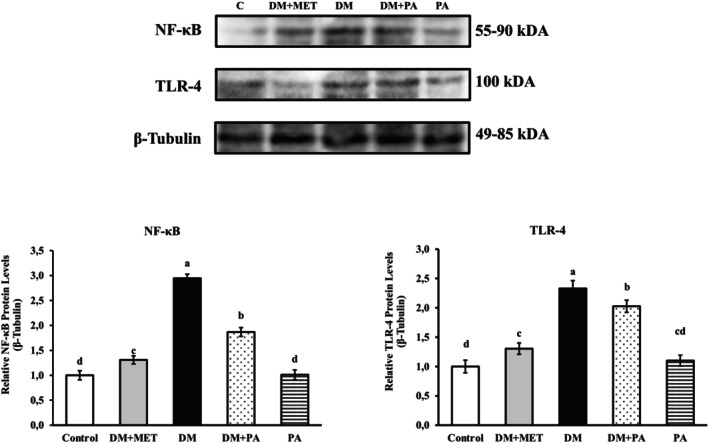
Expression levels of proteins NF‐κB and TLR‐4 in pancreatic tissue of rats. β‐tubulin was used as a housekeeping protein. Densities of NF‐κB and TLR‐4 protein bands in the experimental groups are presented. The bar graph shows as mean ± standard error the (X^¯^±Sx̄) (one‐way ANOVA and Tukey's test) (*p* < 0.001) (*n* = 3). DM, Diabetes; MET, Metformin; PA, *Pediococcus acidilactici* . Significant differences in means are represented by distinct letters, a, b, c, and d.

**FIGURE 2:**
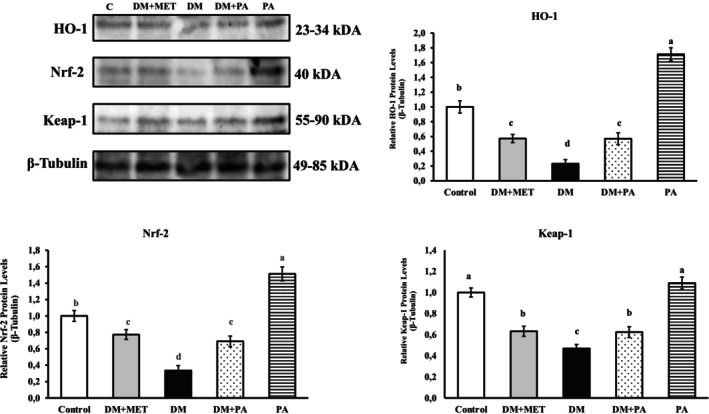
Expression levels of proteins HO‐1, Nrf‐2, and Keap‐1 in pancreatic tissue of rats. β‐Tubulin was used as housekeeping protein. Immunoblots of HO‐1, Nrf‐2, and Keap‐1 from pancreatic lysates. Densities of HO‐1, Nrf‐2, and Keap‐1 protein bands in the experimental groups are presented. The bar graph shows as mean ± standard error the (X^¯^ ± Sx̄) (one‐way ANOVA and Tukey's test) (*p* < 0.001) (*n* = 3). DM, Diabetes, MET, Metformin; PA, *Pediococcus acidilactici* . Significant differences in means are represented by distinct letters, a, b, c, and d.

Western blot results, shown in bands per group (Figures 1 and 2), the expression of NF‐κB and TLR‐4 proteins was the highest in the DM group. The DM group was found to be statistically significant when analyzed with a PA treatment group (*p* < 0.001). So, PA supplementation along with STZ remarkably declined the levels of NF‐κB and TLR‐4 (*p* < 0.001). In the DM group, the level of HO‐1, Nrf‐2, and Keap‐1 was the lowest. The study revealed that the groups treated with PA demonstrated a notable increase in the expression levels of HO‐1, Nrf‐2, and Keap‐1 when compared to the DM group (*p* < 0.001). These findings suggest that PA stimulates the HO‐1, Nrf‐2, and Keap‐1 pathways and plays a significant role in mitigating pancreatic tissue damage associated with diabetes.

### 3.3 Evaluation of Histopathological Analysis

**FIGURE 3:**
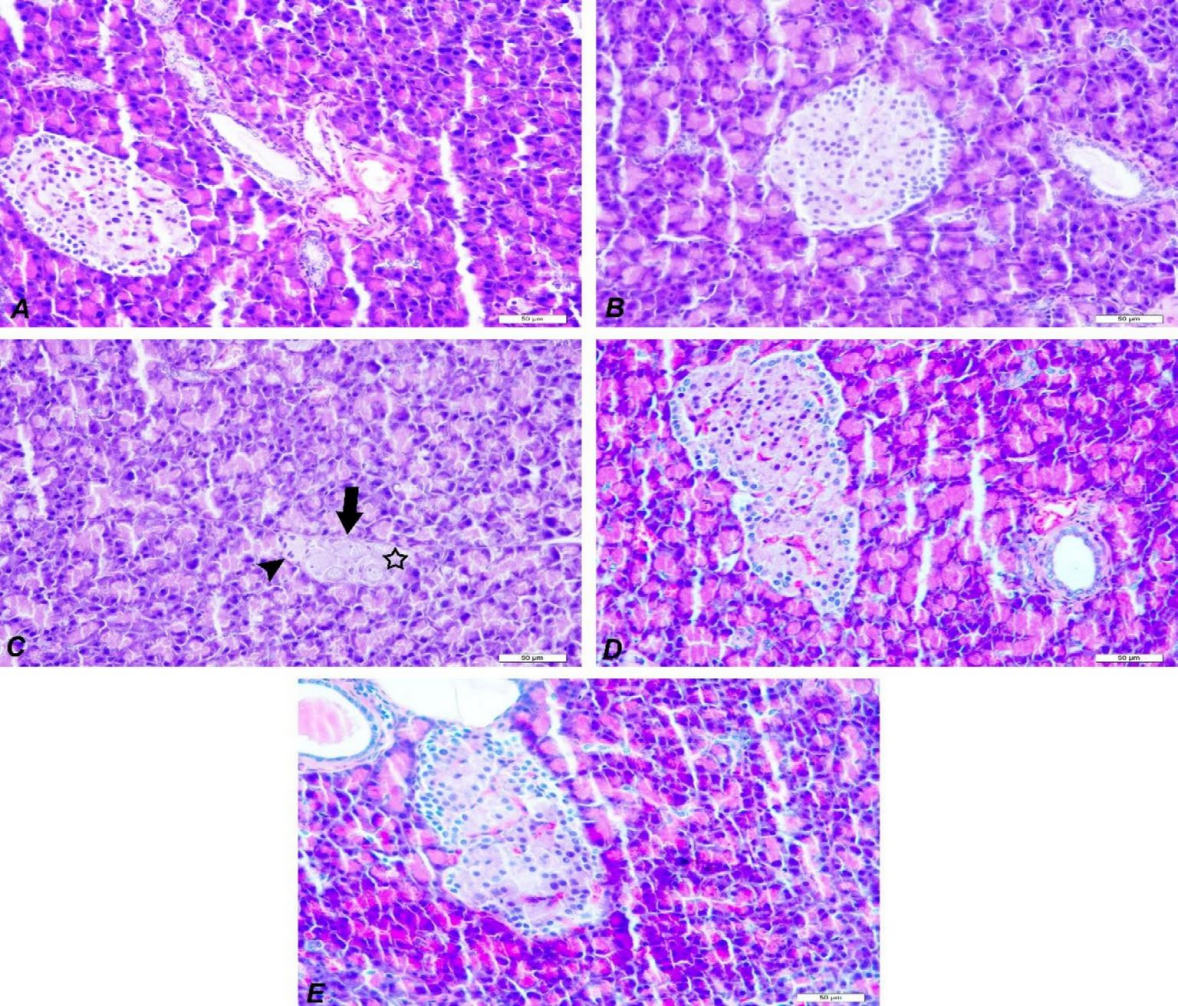
Histopathological appearance of pancreas tissues in all groups. Control group (A): Normal histological structure of the pancreas, DM+MET (B): Improvement in the Langerhans islet structures, DM (C): Severe atrophy in Langerhans islets (arrow), degeneration (arrow head), necrosis (star) in islet cells. DM+PA group (D): Improvement in the Langerhans islet structures. PA group (E): Normal histological structure of the pancreatic tissue. DM, diabetes; MET, metformin; PA, *Pediococcus acidilactici* . H&E, Bar: 50 μm.

In Figure 3A–E, histomorphological observations of pancreatic tissues taken from control, diabetic, and diabetes‐treated rats are presented. The pancreatic tissue of the control group exhibited a histopathologically normal appearance; neither degeneration nor necrosis was observed in the cells of the Langerhans islets (Figure 3A). However, degenerative and necrotic changes were observed in the acinar cells of the Langerhans islets in the DM group, and some of the Langerhans islets were noticed to be irregular and atrophic. In addition, mononuclear cell infiltration consisting mainly of lymphocytes was observed in the DM group (Figure 3C). In the treated groups (DM+MET and DM+PA), degenerative and necrotic changes and inflammatory reactions were significantly reduced. Improvements were found in the structure of the Langerhans islets (Figure 3B,D). Only the histopathological appearance of the pancreatic tissue of the animals in the PA group was quite similar to the control group (Figure 3E).

**FIGURE 4:**
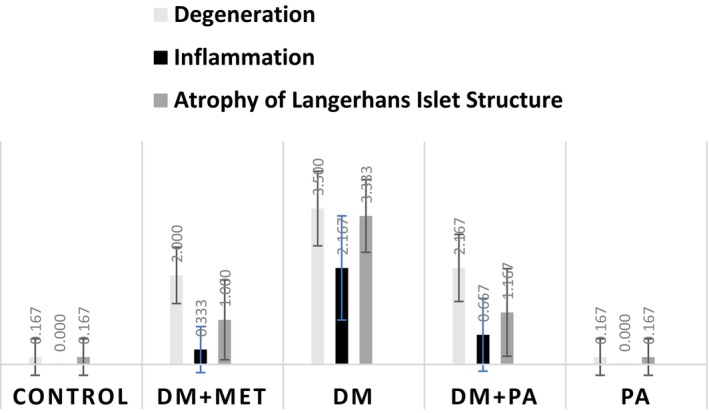
Statistical analysis data of the staining results of histopathological findings (HE) seen in pancreatic tissue. DM, diabetes; MET, metformin; PA, *Pediococcus acidilactici* .

Statistical analysis of the staining results for histopathological findings (HE) in pancreatic tissue revealed significant differences. Degeneration and necrosis, inflammation, and atrophy of Langerhans islet structure were found to be statistically significant (*p* < 0.001). The statistical analysis of staining scores for histopathological findings (HE) in pancreatic tissue is presented in Figure 4.

## 4 Discussion

Metabolic disorders, particularly polygenic diseases like diabetes mellitus, are prevalent worldwide. The number of diabetic patients continues to rise, with the global prevalence among adults reaching 8.8% in 2017. This figure is projected to increase to 9.9% by 2045. In 2017, approximately 424.9 million people were affected by diabetes globally, and this number is expected to rise to around 628.6 million by 2045 (Standl et al. 2019). Diabetes is currently the 7th leading cause of death. The disease is often the result of interactions between genetic and environmental factors, characterized by the absence of hormone secretion and resistance, leading to disturbances in fat, protein, and carbohydrate metabolism (Ramachandiran et al. 2020).

It is necessary to replicate both insulin resistance and β‐cell dysfunction to induce T2DM in experimental animals. The administration of streptozotocin (STZ) can produce this result. DNA breakage and permanent β‐cell damage result from the methyl‐nitrosourea moiety of STZ binding to the GLUT2 transporter on pancreatic β‐cells. This process is similar to the pathophysiology of type 2 diabetes in adult rats and adds to insulin resistance. However, several studies have shown that high dosages of STZ (60–70 mg/kg) may result in substantial β‐cell death, which is more akin to type 1 diabetic mellitus (T1DM) than type 2 diabetes (Goyal et al. 2016; Furman 2021). Pancreatic toxicity is a major feature of diabetes (Shimo et al. 2018).

When STZ is administered, pancreatic β‐cells are destroyed, which significantly impairs glucose consumption and causes energy shortages. Rats lose weight as a result of this metabolic imbalance, which forces the body to use different energy sources, mainly through the catabolism of muscle proteins and adipose tissue (Cruz et al. 2021; Ghasemi and Jeddi 2023). The primary goal of diabetes treatment is to regulate blood glucose levels effectively. This is achieved through insulin, oral antidiabetic drugs, medical nutrition therapy, and exercise. Various strategies are being developed for medical nutrition therapy as part of this multifaceted approach. In particular, herbal nutrients, probiotics, prebiotics, and bioactive compounds with antidiabetic, antioxidative, and anti‐inflammatory properties are under investigation (Krawczyk et al. 2023; Aryal et al. 2024).

Treatment with PA prevented weight loss, suggesting its protective effects against necrotic damage, insulin resistance, proteolysis and lipolysis. Additionally, the significant reduction in glucose and HbA1c levels in PA‐treated animals highlights its hypoglycemic potential. The observed increase in serum insulin levels following PA administration may be attributed to glucose‐stimulated insulin secretion and the modulation of intracellular calcium mobilization (Drzazga et al. 2020). Type‐2 diabetes mellitus (T2DM) is a severe chronic metabolic disorder associated with insulin resistance (impaired cellular response) and progressive pancreatic β cell dysfunction, contributing to elevated blood glucose levels (Himanshu et al. 2020). Widodo et al. (2023), investigated the effects of fermented skim milk containing *Pediococcus pentosaceus* strain M103 and *Pediococcus acidilactici* BE in rats induced with diabetes using STZ and fed a high‐fat pellet diet. Their findings indicated that milk fermented with the *P. acidilactici* BE strain significantly reduced serum glucose levels in diabetic rats. These results support the study's conclusions.

Diabetes occurs when insulin fails to regulate glucose metabolism properly, leading to elevated blood glucose levels and associated health complications. Chronic hyperglycemia in diabetes mellitus increases the production of reactive oxygen species, triggering oxidative stress and damaging pancreatic beta cells, which further disrupts insulin production (Widodo et al. 2023).

Insulin resistance is thought to be mostly influenced by the gut microbiota's makeup, which is regulated by nutrition (Zhang and Zhang 2013). According to Moon et al. (2014), the probiotics *P. acidilactici* DSM 20284 and PA strain M76 can lower cholesterol levels in vivo. Prior research has demonstrated that *P. pentosaceus* strain M103 and PA strain BE can acidify milk while preserving its favorable physicochemical and microbiological characteristics. In diabetic rats, these probiotics might also have an impact on blood glucose levels. To the best of our knowledge, however, little study has been done on these impacts (Widodo et al. 2017).

Our research shows that oxidative stress is linked to STZ‐induced diabetes, as seen by a notable rise in MDA levels. The fact that hyperglycemia increases the generation of reactive oxygen species (ROS) helps to explain this. On the other hand, the higher MDA levels were considerably reversed by the PA probiotic strain.

As shown in Table 1, endogenous enzymatic antioxidant indicators (CAT, SOD) were identified. PA was found to reduce oxidative stress through these endogenous enzymes because they are crucial in reducing oxidative stress brought on by DM (Sheweita et al. 2020). The diabetic group in this study had low activities of CAT and SOD; nevertheless, probiotic PA treatment significantly raised antioxidant levels. This could be explained by the impairment of lipid peroxidation pathways caused by probiotic treatment. Antioxidant enzyme activity was shown to be decreased in DM rats in the current investigation. This result adds credence to earlier research indicating a link between diabetes and compromised antioxidant defenses (Aluwong et al. 2016).

In addition to oxidative stress, chronic inflammation and endothelial dysfunction are the main cellular changes that play a role in the pathogenesis of DM (Méndez‐Morales et al. 2022). Among the inflammatory actors involved in the pathogenesis and progression of a wide variety of inflammatory disorders, TLR4 is a member of the pattern recognition receptors that are critical in the body's inflammatory responses and immunity. There are studies reporting that TLR4 plays a role in maintaining inflammation during diabetic complications (Dasu et al. 2012). Research demonstrating Nrf‐2's function as the body's defensive mechanism against both internal and external stressors has demonstrated that Nrf‐2 is able to assess the level of oxidative stress in cells and, by controlling the redox balance of those cells, lessen the harm that oxidative stress causes. When Nrf‐2 and HO‐1 bind to antioxidant response elements in promoter regions, they activate several downstream genes and increase the synthesis of antioxidant enzymes. According to our findings, it was determined that Nrf2‐ and HO‐1 protein levels significantly decreased in the pancreas of DM rats, and Nrf‐2 and HO‐1 protein levels decreased significantly after PA treatment. Based on these findings, we propose that PA alleviates pancreas damage in DM rats by activating the Nrf‐2 antioxidant signaling pathway. This activation promotes the expression of downstream genes and enhances the production of antioxidant enzymes, thereby improving the pancreas's antioxidant capacity.

Finally, it has been found that the HO‐1 gene is an essential gene with antioxidant capacity (Huang and Fang 2020). In this study, a significant reduction in HO‐1 protein expression was seen in the diseased groups (Figure 2), indicating that the antioxidant activity of HO‐1 is overwhelmed by the oxidative stress brought on by diabetes (Drummond et al. 2019). However, it was found that daily administration of PA for 21 days considerably reversed the elevated levels of HO‐1 in PA groups.

Oxidative stress is regarded as a major factor contributing to tissue injury and diabetic complications. It serves as a crucial indicator for assessing the health of beta cells in the islets of Langerhans (Leenders et al. 2021). In the histopathological findings obtained in the study, the islets of Langerhans were disorganized and atrophied due to degenerative and necrotic alterations in the Langerhans cells, with occasional degeneration of acinar cells. Additionally, mononuclear cell infiltration, predominantly composed of lymphocytes, was observed in the DM group. In the DM+PA group, it was noted that the Langerhans cell degeneration considerably slowed down, and the signs of necrosis disappeared.

The administration of *Pediococcus acidilactici* to STZ‐induced diabetic rats had an impact on the pancreas, raising insulin levels while lowering glucose and HbA1c levels and producing hypoglycemic effects. Applying PA reduced lipid peroxidation and cytokine levels while enhancing SOD and CAT activities. It was found that diabetes produced oxidative damage by raising cytokine levels (TNF‐α and IL‐1β). Furthermore, since no prior research has investigated the impact of PA on NF‐κB, Tlr‐4, and the HO‐1, Nrf‐2, and Keap‐1 signaling pathways in STZ‐induced diabetic pancreatic tissue, this study serves as a pioneering contribution to the literature. Given these findings, PA may be considered a potential therapeutic agent for managing DM. Additionally, further research is warranted to explore its role in other metabolic diseases and diabetic complications.

## Author Contributions

**Kubra Asena Terim Kapakin:** methodology, data curation. **Tuba Karaarslan:** methodology, data curation. **Betul Apaydin Yildirim:** methodology, conceptualization, formal analysis, writing – original draft, writing – review and editing, investigation, supervision, resources, data curation. **Enes Tercanli:** methodology, conceptualization, investigation, writing – original draft, resources, supervision, writing – review and editing. **Mustafa Atasever:** methodology, data curation.

## Funding

The authors have nothing to report.

## Ethics Statement

Animal trials were conducted at the Atatürk University Medical Experimental Research Center. This study was approved by Atatürk University Animal Experiments Local Ethics Committee (E‐36643897‐000‐2,400,417,803).

## Conflicts of Interest

The authors declare no conflicts of interest.

## Data Availability

The data that support the findings of this study are available on request from the corresponding author. The data are not publicly available due to privacy or ethical restrictions.
